# 483. Disease Severity and Clinical Manifestations of SARS-CoV-2 Infection Among Infants Over the First Year of the Pandemic in Canada

**DOI:** 10.1093/ofid/ofab466.682

**Published:** 2021-12-04

**Authors:** Pierre-Philippe Piché-Renaud, Luc Panetta, Daniel Farrar, Charlotte Moore Hepburn, Olivier Drouin, Fatima Kakkar, Shaun Morris

**Affiliations:** 1 The Hospital for Sick Children, Toronto, Toronto, Ontario, Canada; 2 Hôpital Femme Mère Enfant, Lyon, France, Lyon, Auvergne, France; 3 Centre for Global Child Health, Toronto, Ontario, Canada; 4 Hospital for Sick Children, Toronto, Ontario, Canada; 5 CHU Sainte-Justine, Montreal, Quebec, Canada; 6 Hospital for Sick Children, University of Toronto, Toronto, Ontario, Canada

## Abstract

**Background:**

There is limited data on outcomes of SARS-CoV-2 infection among infants (< 1 year of age). In the absence of any approved vaccines for infants, understanding the risk factors for hospitalization and severe disease from COVID-19 in this age group will help inform clinical management and targeted public health interventions. The objective of this study was to describe the clinical manifestations, disease severity, and risk factors for hospitalization among infants with SARS-CoV-2 infection in Canada.

**Methods:**

This is a nationwide prospective observational study using the infrastructure of the Canadian Paediatric Surveillance Program. All cases of infants aged < 1 year of age with microbiologically confirmed SARS-CoV-2 infection were reported from April 8^th^ 2020 to May 11^th^ 2021, and classified by disease severity, and primary cause of hospitalization. Logistic regression was performed to identify risk factors for hospitalization and severe disease.

**Results:**

A total of 393 cases were reported, including 229 (58.3%) non-hospitalized and 164 (41.7%) hospitalized infants. The most common symptoms included fever (63.4%), runny nose (45.0%), cough (35.1%) and decreased oral intake (24.9%). Significant risk factors for hospitalization included younger age and presence of comorbid conditions (excluding prematurity), as shown in the Table. Among hospitalized infants, 108 (65.9%) were admitted because of COVID-19-related illness, and 52 (31.7%) were admitted for reasons other than COVID-19. A total of 31 (7.9%) infants developed severe or critical disease. Risk factors for severe disease included prematurity and younger age (Table).

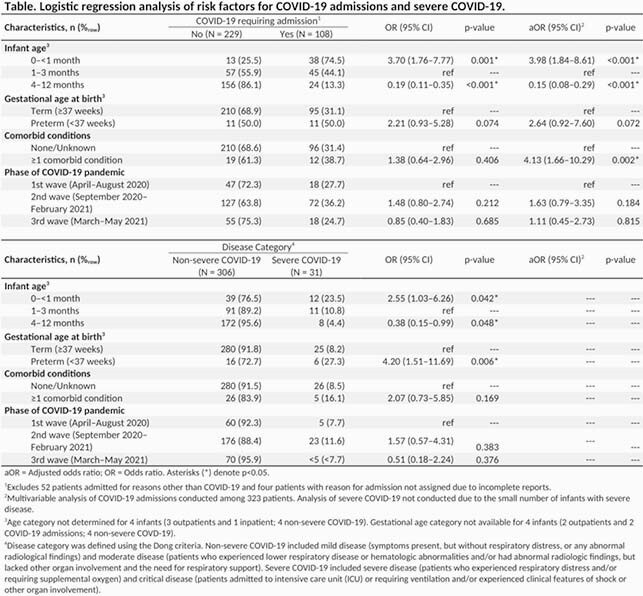

**Conclusion:**

We describe one of the largest cohort of infants with SARS-CoV-2 infection. Severe disease in this age group is uncommon, with younger age and prematurity being significant risk factors for severe COVID-19.

**Disclosures:**

**Pierre-Philippe Piché-Renaud, MD**, **Pfizer Global Medical Grants (Competitive grant program**) (Research Grant or Support, Investigator-led project on the impact of COVID-19 on routine childhood immunizations) **Olivier Drouin, MDCM MsC MPH**, **Covis Pharma** (Research Grant or Support) **Shaun Morris, MD, MPH, DTM&H, FRCPC, FAAP**, **GSK** (Speaker’s Bureau)**Pfizer** (Advisor or Review Panel member)**Pfizer** (Grant/Research Support)

